# Stochastic modelling of biochemical systems of multi-step reactions using a simplified two-variable model

**DOI:** 10.1186/1752-0509-7-S4-S14

**Published:** 2013-10-23

**Authors:** Qianqian Wu, Kate Smith-Miles, Tianshou Zhou, Tianhai Tian

**Affiliations:** 1 School of Mathematical Sciences, Monash University, VIC 3800 Melbourne, Australia; 2 School of Mathematics and Computational Sciences, Sun Yet-Sen University, Guangzhou 510275, P.R. China

## Abstract

**Background:**

A fundamental issue in systems biology is how to design simplified mathematical models for describing the dynamics of complex biochemical reaction systems. Among them, a key question is how to use simplified reactions to describe the chemical events of multi-step reactions that are ubiquitous in biochemistry and biophysics. To address this issue, a widely used approach in literature is to use one-step reaction to represent the multi-step chemical events. In recent years, a number of modelling methods have been designed to improve the accuracy of the one-step reaction method, including the use of reactions with time delay. However, our recent research results suggested that there are still deviations between the dynamics of delayed reactions and that of the multi-step reactions. Therefore, more sophisticated modelling methods are needed to accurately describe the complex biological systems in an efficient way.

**Results:**

This work designs a two-variable model to simplify chemical events of multi-step reactions. In addition to the total molecule number of a species, we first introduce a new concept regarding the location of molecules in the multi-step reactions, which is the second variable to represent the system dynamics. Then we propose a simulation algorithm to compute the probability for the firing of the last step reaction in the multi-step events. This probability function is evaluated using a deterministic model of ordinary differential equations and a stochastic model in the framework of the stochastic simulation algorithm. The efficiency of the proposed two-variable model is demonstrated by the realization of mRNA degradation process based on the experimentally measured data.

**Conclusions:**

Numerical results suggest that the proposed new two-variable model produces predictions that match the multi-step chemical reactions very well. The successful realization of the mRNA degradation dynamics indicates that the proposed method is a promising approach to reduce the complexity of biological systems.

## Background

The advances in systems biology have raised the importance of quantitative methods for studying various systems in molecular biology. In recent years, various research methods, including mathematical modeling, statistical analysis, computer simulation and visualization, have been employed to investigate the dynamic or statistical properties of regulatory networks. In particular, mathematical models have been widely used to describe the dynamics of complex systems inside the cell, including genetic regulatory networks, cell signalling transduction pathways and metabolic pathways [1][2]. However, these substantial progresses have further raised a number of fundamental and challenging issues that require to be addressed imperatively.

One of the major challenges in systems biology is how to use simple mathematical models to describe complex biological systems. To address this issue, a number of modelling techniques have been designed. Among them, a widely used approach is to use one-step reaction to represent multi-step reactions, which is also called slow reaction. This technique is very important because recent theoretical and experimental studies have shown that a wide variety of biochemical events involve multi-step reactions [3]. Perhaps the most important example of multi-step reactions is transcriptional and translational processes that produce mRNA transcripts and proteins, respectively. Other examples include molecules (e.g. mRNA and protein) degradation and telomere length shortening processes. In fact, the process of multi-step reactions also exists in other areas such as organic chemistry and biophysical chemistry [4][5]. Therefore the major aim of this research work is to design simplified models to accurately characterize biological systems with multi-step reactions.

A widely used approach to simplify multi-step chemical reactions in the literature is to use one-step reaction. For example, the degradation process of mRNA or protein has been modelled by a first order reaction. However, since the one-step reaction cannot provide consistent description of the multi-step reactions, chemical reactions with time delay have been designed recently to model the multi-step chemical events more accurately [6][7][8][9]. Another important factor is noise in biological networks that may influence the system dynamics substantially. The deterministic modelling methods, which approximate molecular numbers using continuous concentrations [10][11], may not be appropriate to describe systems that contain species with small population numbers. To model stochastic systems more accurately, there are a few other ways. For example, we can use discrete Markov processes where the density of states of a well-stirred chemical reaction system at each time point can be represented by the chemical master equation (CME) [12][13]. One of the most well-known methods is called the stochastic simulation algorithm (SSA), which is a statistically exact method for simulating trajectories of the CME as the system evolves in time [14].

Furthermore, to deal with the intrinsic noise in reactions with time delay, the delay stochastic simulation algorithm (DSSA) was designed by introducing time delay into the SSA [15][16]. Unlike the SSA, which assumes that biochemical reactions are instantaneous and independent, the DSSA characterizes chemical systems that contain both fast and slow reactions. This delayed modelling approach has been applied to many physical and biological systems [16]. The DSSA was also extended to describe chemical events that have multiple delays or stochastic delay that follows a given probabilistic distribution [17][18]. In recent years, the DSSA has been widely used to simulate the dynamics of genetic regulatory networks and cell signalling pathways [7][19][20][21][22]. In addition, a number of effective simulation methods have been proposed to reduce the huge computing load of the DSSA [23][24][25][26]. Recently the work done by Luis Mier-y-Terán-Romero et al. opened some new aspects for the application of time delays in biological systems. Time delay may not be a constant that was assumed before [27]. Other modelling techniques proposed recently include the slow-scale linear noise approximation and stochastic quasi-steady-state assumption [28][29]. Most recently a new modelling approach has been proposed to simulate chemical reaction systems with memory reactions [30].

The degradation process of mRNA molecules is an important step in the regulation of gene expression, which also represents a typical system with multi-step reactions [31]. Although the mechanisms of mRNA degradation have been studied extensively during the last ten years, there are still a number of open problems with respect to the function of enzymes, structure of pathways and role of P-bodies, etc. in the regulation of mRNA degradation [32][33][34]. A major step in the quantitative study of mRNA degradation was the development of mathematical models based on the detailed chemical processes. A linear multi-component model was designed to investigate the nonsense-mediated decay of mRNA molecules in yeast [35][36]. This deterministic model for mRNA degradation process consists of 23 first-order reactions that describe transcription, translocation, ploy(A) shortening, decapping and digestion process. Computer simulations suggested that the widely used concept of half-life underestimated the averaged life-span of mRNA molecules; however, it is still a major factor that determines the life-span of different steps in the degradation pathway. In addition, robustness analysis showed that the change of degradation rate constant led to large variations of mRNA copy numbers. To interpret the complexity of mRNA degradation in a simpler manner, we proposed a multi-step reaction model using a chain of 11 chemical reactions, which gave very good approximation to the detailed one [37].

Chemical reactions with time delay has been used to further simplify mathematical models of mRNA degradation. Here time delay represents the time required in the multi-step reactions except the first reaction [37]. This simplified model was also extended to using stochastic time delay. However, numerical results showed that these first-order reaction models with delay did not give good approximation to the detailed degradation process [37]. Instead of using time delay to represent the missing intermediate reactions in the multi-step reaction, we recently proposed a new modelling approach by introducing a novel concept, namely the length of a molecule indicating its location in the multi-step reactions. Deterministic models using ordinary differential equations have been used to find the optimal value in a non-linear probability function [38]. However, it is still a challenge to apply this concept to stochastic models that are much more important than deterministic models for chemical reaction systems. Thus this work further validates the proposed model using stochastic simulations. We first introduce a new stochastic modelling method with two variables for describing chemical events with multi-step reactions, and then propose a stochastic simulation algorithm to numerically calculate the probability of the firing of the last reaction in the multi-step events. The efficiency and accuracy of the proposed method are examined by studying the mRNA degradation process of gene PRL30 based on experimental data.

## Results and discussion

### A new two-variable model

The starting-point of this research work is the chemical events with multi-step reactions. Using the notation proposed in [3], we consider the following chemical reactions

(1) $$B_{1}\overset{k_{1}}{\to }\;B_{2}\overset{k_{2}}{\to }\;B_{3}\overset{k_{3}}{\to }\cdots \;\overset{k_{n}}{\to }\;B_{n}\;\overset{k_{n}}{\to }\;P.$$

where *B_i _*are molecular species and *k_i _*rate constants. It is assumed that each molecule in the system will eventually turn to the product *P *or degrade if *P *= (). During this process, each molecule will pass through a number of states *B*_1_, *B*_2_, . . . , *B_n _*via the multi-step reactions.

When the number of reaction step *n *is large, we need to design a smaller scale model to simplify the multi-step reactions. We first consider the total number of molecules in the system, defined by

(2) $$X=\overset{n}{\underset{i=1}{\sum }}\left[B_{i}\right].$$

Here we introduce a new concept to describe the system state. The number of reactions for a molecule to reach the product *P *is termed as the length of that molecule. Thus the length of molecule *B_i _*is (*n *− *i *+ 1) and the total length of the molecules in the system is

(3) $$L=\overset{n}{\underset{i=1}{\sum }}\left(n-i+1\right)\left[B_{i}\right].$$

According to the total molecule number, chemical reactions in the system can be classified into two groups. If one of the first (*n *− 1) step reactions occurs, namely $B_{i}\overset{k_{i}}{\to }\;B_{i+1}$, the total number of molecules *X *is unchanged but the total length *L *is decreased by one,

(4) (X,L)→(X,L-1).

However, if the last reaction $B_{n}\overset{k_{n}}{\to }\;P$ fires, both the total number and total length will decrease by one,

(5) (X,L)→(X-1,L-1).

In this work we use reactions (4) and (5) to design the two-variable reaction model.

**Figure 1:**
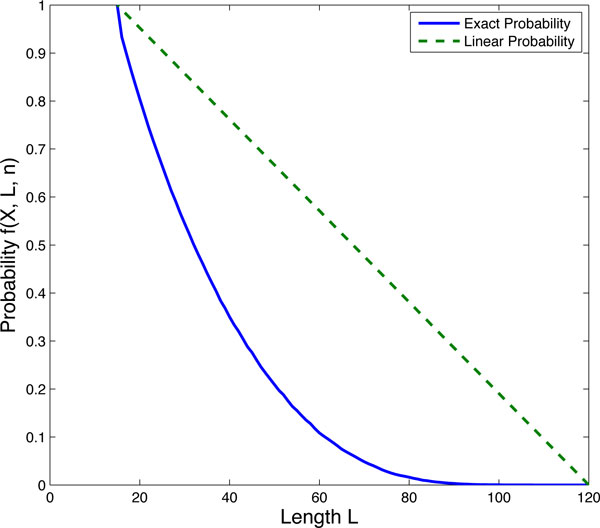
**The probability for the firing of the last reaction $B_{n}\overset{k_{n}}{\underset{}{\to }}P$**.

The key question now is how to determine whether reaction (4) or (5) will fire if one of the reactions in the multi-step process (1) happens. We denote the probability for the degradation of one molecule, namely the firing of reaction (5), as *f *(*X, L, n*), and then the corresponding probability for reaction (4) as 1 − *f *(*X, L, n*). It is clear that, when all molecules are of full length (*X *= *nL*), the probability of *f *is zero; while when *X *= *L*, the probability is one. For the molecules with other lengths, we developed an algorithm, namely Algorithm I in the Method section, to calculate the probability of molecule degradation. With the help of this algorithm, we numerically calculated the exact probability *f *(*X, L, n*) using *n *= 8 and *X *= 15 as an example. The probability is represented in Figure 1 as the solid line.

Next we find an appropriate probability function to approximate the calculated curve in Figure 1. Note that the total length *L *of *X *molecules satisfies *X *≤ *L *≤ *nX *. When *L *= *X *, all molecules have length 1, the probability of firing of the last step reaction is 1, i.e. *f *(*X, L, n*) = 1; when *L *= *nX *, all molecules have length *n*, there is no chance for the final reaction to occur in the next step, i.e. *f *(*X, L, n*) = 0. Therefore we suggested a probability function to approximate the curve in Figure 1 in the following format:

(6) $$f\left(L,X,n\right)=1-\frac{L-X}{X\left(n-1\right)}.$$

The approximated probability through the proposed function (6) is plotted as the straight dashed line in Figure 1. It shows that the approximated values are not close to the exact probability values, and the exact probability curve is in a quadratic-like form. Hence, instead of using a linear probability *f *in terms of *X , L *and *n *(6), we introduced another parameter *q *into this approximation, and proposed the following two expressions for the probability function *f *in terms of *L, X, n*, and *q*. One candidate is

(7) $$\text{Type}\;\text{I}:\;\;f\left(X,L,n,q\right)=1-{\left(\frac{L-X}{X\left(n-1\right)}\right)}^{q},$$

and the alternative expression is

(8) $$\text{Type}\;\text{II}:\;\;f\left(X,L,n,q\right)={\left(1-\frac{L-X}{X\left(n-1\right)}\right)}^{q}.$$

### Determination of probability function

**Figure 2:**
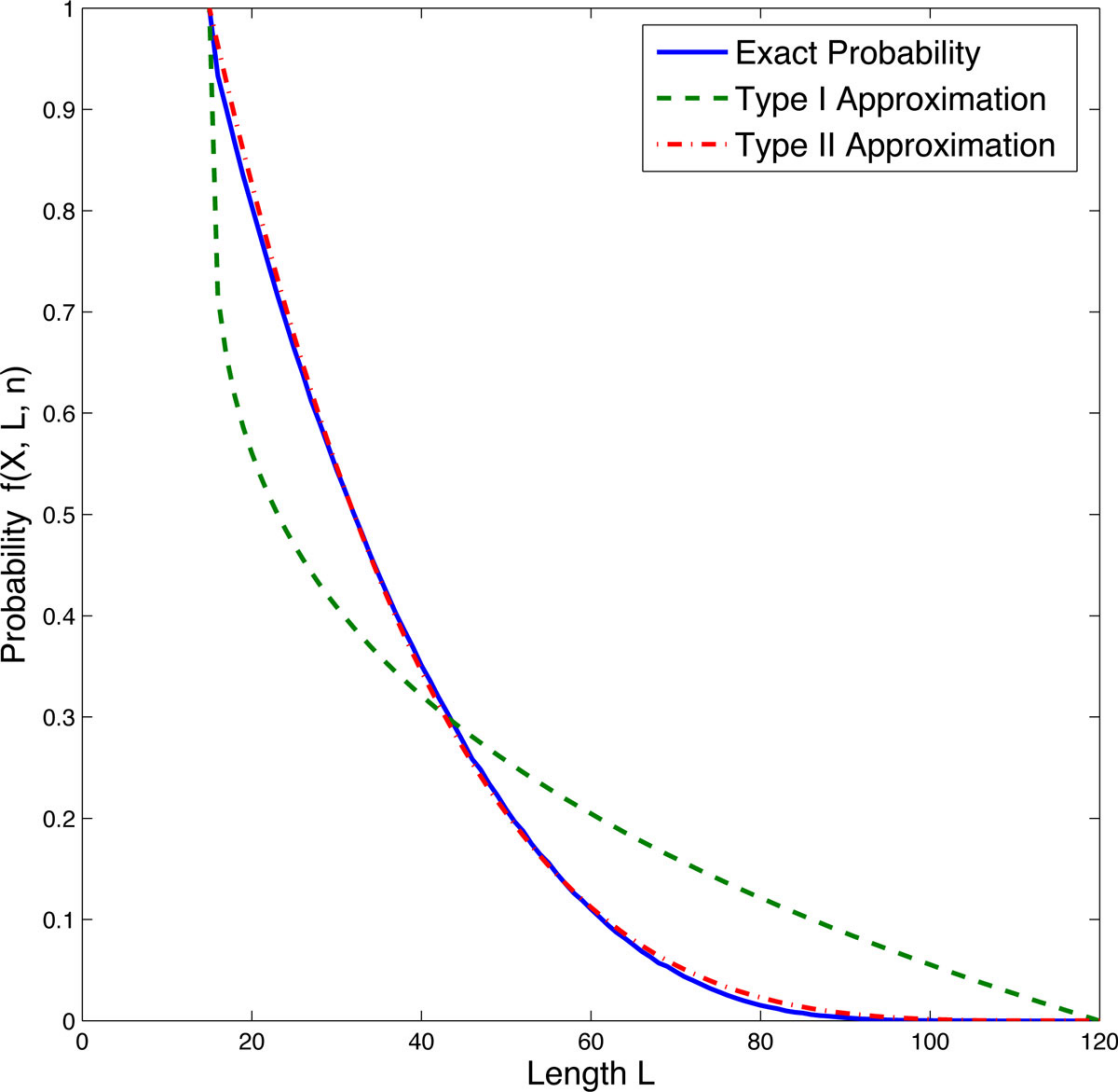
**Simulated exact probabilities and two approximated probabilities for the firing of the last reaction**.

The major work of this research is to select a probability function from (7) and (8) and also search the optimal value of parameter *q *in the probability function. Using Algorithm I in the Method section, we first calculated the probability *f *(*X, L, n, q*) with different values of the total molecule number *X *(*X *= 3 ~ 20), different numbers of reaction step *n *(*n *= 3 ~ 20) and various values of the total length *L *(*L *= *X *~ *nX *). The calculated probability was used as the exact value to search the optimal *q *in the proposed probability functions. To select a better probability function, we used both type I and II functions to calculate the probability *f *(*X, L, n, q*) using the same initial condition (*n *= 8 and *X *= 15) but different values of *q *(*q *= 0.01 ~ 15) in a step size of 0.01. By searching for the smallest difference between the exact probability values and those obtained from approximated functions with different *q*, the optimal values of *q *for two approximations were achieved which are 0.27 and 3.91 respectively in this particular case. The exact and approximated probability values are shown in Figure 2. We found that the type II approximation is closer to the exact probabilities than the type I approximation. Then we only used the probability function (8) for the following studies.

**Figure 3:**
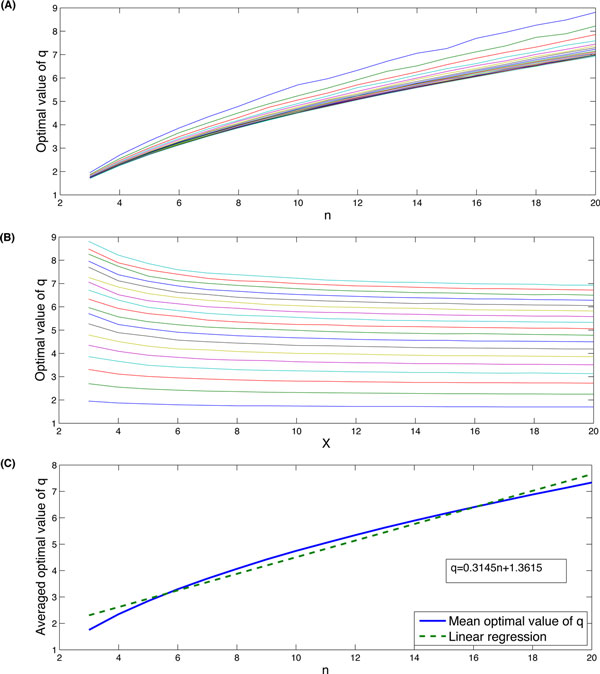
**Simulation results from probability approach**. (A) the optimal values of *q *with different *n*; (*B*) the optimal values of *q *with different *X *; (*C*) the averaged optimal values of *q *against *n*.

To establish a more general formula for defining *q *under different conditions of *X, L, n*, we extended the simulations to various initial conditions of *n, X *both varying from 3 to 20 together with different values of *q*. The optimal *q *values acquired under these conditions are illustrated by Figure 3 (*A*) and (*B*). Figure 3 (*A*) shows that when *n *increases, the optimal value of *q *increases for a fixed *X *value; while Figure 3 (*B*) indicates that there is no significant variation for the optimal *q *when *X *increases for a fixed *n *value. Therefore, we calculated the averaged optimal *q *values under various value of *n *for each given *X*. A plot of this averaged optimal $\bar{q}$ against *n *is shown in Figure 3 (*C*). We suggested a linear relationship between *n *and *q*. A linear regression analysis suggested that this relationship is

(9) $$\bar{q}=0.3146n+1.3615.$$

**Figure 4:**
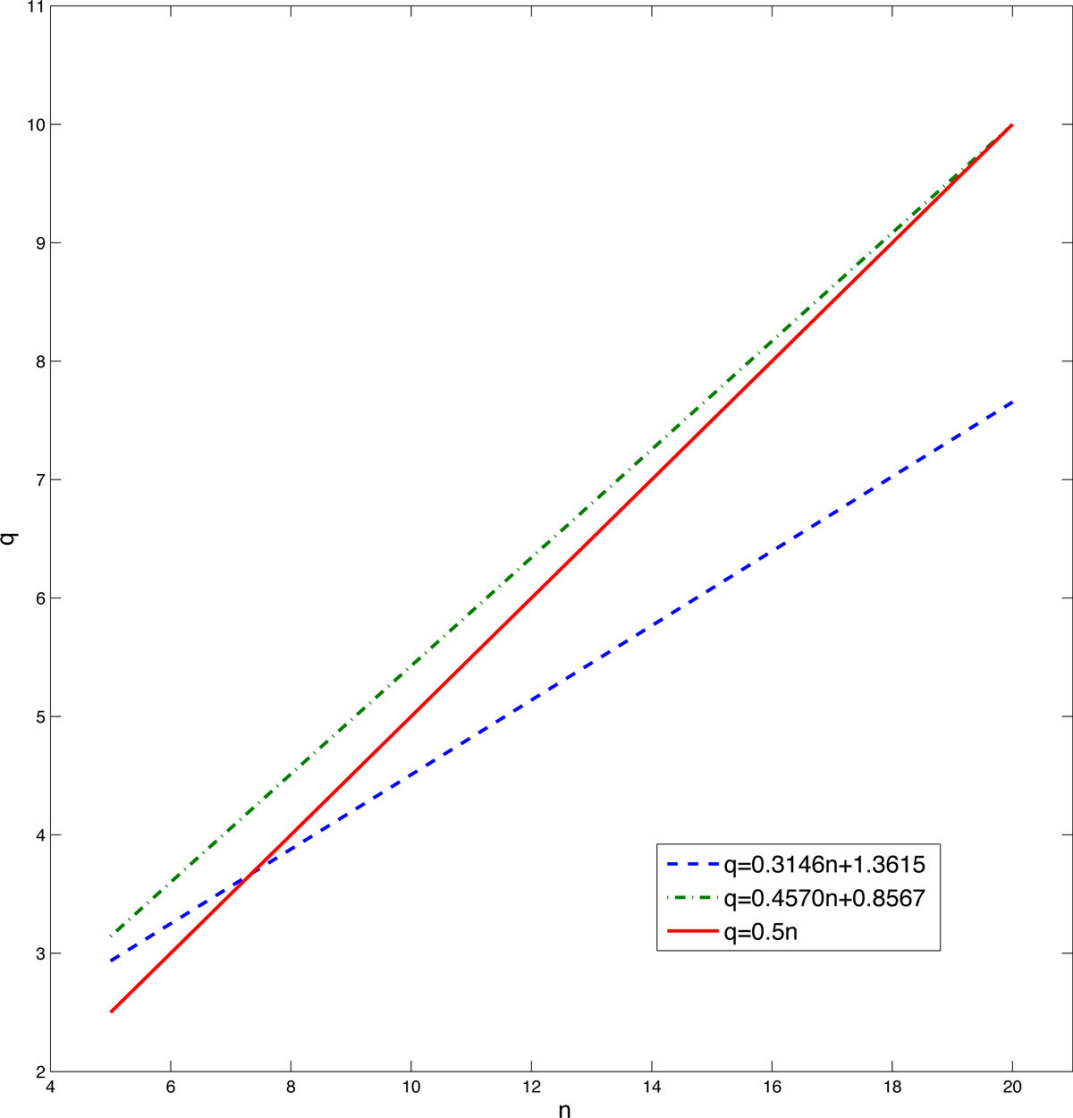
**Relationship between *n ***and ***q***. Dashed-line: estimated relationship from stochastic simulations; dash-dot-line: relationship derived from the ODE model; Solid-line: *q *= 0.5*n*.

We have developed deterministic models of ordinary differential equations (ODEs) based on the multi-step reactions (1) and the two-variable model (4, 5) [38]. Simulation results of the deterministic models gave some similar patterns such that the optimal value of *q *increases when the number of chemical reactions *n *increases. The established relationship between the optimal value of *q *and related model parameters, which is also shown in Figure 4, is given by

(10) $$\bar{q}=0.4570n+0.8567.$$

The above equation is slightly different from expression (9). For example, when *n *= 5, the averaged value of optimal *q *is found to be 2.8494 using probability simulation while it is 3.06 from the ODE simulation. The possible reason of the difference is that the ODE model is not the best approach for describing chemical reaction systems with molecules of small copy numbers and some model errors may arise from the ODE simulations. A combination of regression analyses is shown in Figure 4.

Even with the different formulations for the optimal *q *values, we still find that the ODE method confirms the conclusion derived from stochastic simulations. Based on both stochastic and deterministic simulations, we found that the value of *q *is associated with the number of reaction step *n*, but not connected to the total molecule number *X*. Our results also suggested that, when the value of *q *approaches the optimal one, simulation error of the two-variable model using *q *is very close to that using the optimal *q *value. Using the function derived from stochastic simulations, the probability function of molecule degradation is given by

(11) $$f\left(L,X,n\right)={\left(1-\frac{L-X}{X\left(n-1\right)}\right)}^{0.3146n+1.3615}.$$

Using this probability function, we designed an algorithm, namely Algorithm II in the Method section, to simulate the two-variable reaction model based on the SSA.

### mRNA decay dynamics: case study for gene RPL30

In this section, we apply the established theory in the previous section to study the dynamics of mRNA degradation. Here we use gene ribosomal protein L30 (*RPL30*) as the test system with a dataset generated from experiments. In these experiments, two constructs of *RPL30 *were used to demonstrate the decay kinetics of the mRNA transcripts [39]. The first construct ("construct *A*") contains the *ACT1 *UAS (upstream activating sequence), and the other ("construct B") contains the *RPL30 *UAS. The mRNA molecule decay dynamics was monitored after blocking transcription by using drug 1,10-phenanthroline [39]. Thus we assumed that there was no further transcription during the monitoring process. The decay dynamics was normalized by the *RPL30 *transcript level at time zero (namely before adding the drug), which was set to 100%. Using the endogenous *RPL30 *mRNA levels obtained from the two construct [39], we first used the one-step differential equation model

(12) $$\frac{dX}{dt}=-kX$$

to simulate the decay dynamics [40].

**Figure 5:**
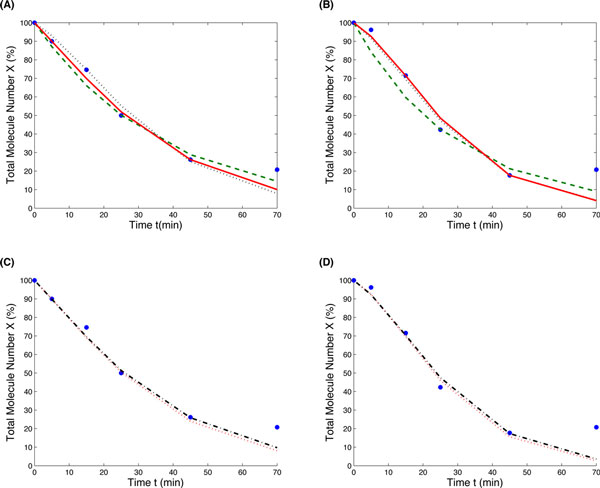
**Simulated mRNA degradation dynamics using the estimated model parameters**. (*A*) Deterministic simulations for mRNA numbers from the *ACT1 *construct (green dash-line: the one-step model (*k *= 0.0276), red solid-line: the two-variable model with the optimal initial length (*k *= 0.112, *L *= 371), black dot-line: the two-variable model with the averaged initial length $L=\frac{nX}{2}$, blue dots: experimental data); (*B*) Deterministic simulations for mRNA numbers from the *RPL30 *construct (green dash-line: the one-step model (*k *= 0.0343), red solid-line: the two-variable model with the optimal initial length (*k *= 0.167, *L *= 473), black dot-line: the two-variable model with the averaged initial length $L=\frac{nX}{2}$ (*k *= 0.161), blue dots: experimental data); (*C*) Stochastic simulations of the two-variable model for the *ACT1 *construct (red dot-line: initial *X*_0 _= 5, *k *= 0.115, *L *= 19, black dash-dot-line:initial *X*_0 _= 10, *k *= 0.111, *L *= 37, blue dots: experimental data); (*D*) Stochastic simulations of the two-variable model for the *RPL30 *construct (red dot-line: initial *X*_0 _= 5, *k *= 0.171, *L *= 24, black dash-dot-line:initial *X*_0 _= 10, *k *= 0.166, *L *= 47, blue dots: experimental data).

Figure 5 (*A*) and (*B*) show that the one-step model failed to describe the dynamics of the first 25 minutes accurately. The simulated mRNA levels are always smaller than the experimental observation.

To model mRNA degradtion, Cao and Parker proposed a multi-component model that includes mRNA transcript synthesis, mRNA translocation, poly(A)-shortening process, and terminal deadenylation [35]. We have proposed a simplified model by putting a number of terminal deadenylation reactions into a single one [37]. This simple model is a typical multi-step reaction process. In this model, mRNA transcript is synthesized by a zero-order reaction *S*_1_, then mRNA molecules translocate from the nucleus to cytosal via reaction *S*_2_. The mRNA molecules in the cytosol produce proteins by the translational process, and in the meantime, the length of mRNA begins to decrease via a number of poly(A)-shortening reactions *S*_3_, . . . , *S*_9_. The final reaction in this process is the further exonucleolytic degradation *S*_10_, which is regarded as the degradation reaction in this work, since the fragment product (FG) has no function to produce protein molecules.

Based on the reactions listed in Table 1 and rate constants, the propensity functions of these reactions are listed below.

**Table 1:** Reactions and kinetic rates of the simplified stochastic model.

	Reaction	Rate constant *s_i_*	Comment
*S*_1_	*DNA *→ *A*	*s*_1_	transcription
*S*_2_	*A *→ *B*	*s*_2_	transport
*S*_3_	*B *→ *BC*1	*s*_3_	full-length 70A-60A
*S*_4_	*BC*1 → *BC*2	*s*_4_	full-length 60A-50A
*S*_5_	*BC*2 → *BC*3	*s*_5_	full-length 50A-40A
*S*_6_	*BC*3 → *BC*4	*s*_6_	full-length 40A-30A
*S*_7_	*BC*4 → *BC*5	*s*_7_	full-length 30A-20A
*S*_8_	*BC*5 → *BC*6	*s*_8_	full-length 20A-10A
*S*_9_	*BC*6 → *BC*7	*s*_9_	full-length 10A-0A
*S*_10_	*BC*7 → *FG*	*s*_10_	fragment production

The rate constants ***s_i _***are in the unit of 1/sec.

(13) $$\begin{array}{ccc} \text{Reaction} & \text{ Propensity function} & \\ DNA\overset{s_{1}}{\to }A & a_{1}=s_{1},\\ A\overset{s_{1}}{\to }B & a_{2}=s_{2}.\left[A\right], & \\ B\overset{s_{3}}{\to }BC1 & a_{3}=s_{3}.\left[B\right],\\ BC1\overset{s_{4}}{\to }BC2 & a_{4}=s_{4}.\left[BC1\right],\\ BC2\overset{s_{5}}{\to }BC3 & a_{5}=s_{5}.\left[BC2\right],\\ BC3\overset{s_{6}}{\to }BC4 & a_{6}=s_{6}.\left[BC3\right],\\ BC4\overset{s_{7}}{\to }BC5 & a_{7}=s_{7}.\left[BC4\right],\\ BC5\overset{s_{8}}{\to }BC6 & a_{8}=s_{8}.\left[BC5\right],\\ BC6\overset{s_{9}}{\to }BC7 & a_{9}=s_{9}.\left[BC6\right],\\ BC7\overset{s_{10}}{\to }FG & a_{10}=s_{10}.\left[BC7\right], \end{array}$$

Following the experimental conditions, it is assumed that *s*_1 _= 0. For simplicity, it is assumed that *s*_2 _= . . . = *s*_10_. When using the two-variable model to study the mRNA degradation process, we write the total copy number *X *and total length of mRNA molecules *L *as

$$\begin{array}{c} X=\left[A\right]+\left[B\right]+\left[BC1\right]+\cdots +\left[BC7\right],\\ L=9\left[A\right]+8\;\left[B\right]+7B\left[C1\right]+\cdots +\left[BC7\right]. \end{array}$$

Here we put the mRNA synthesis as a separate reaction. Then the remaining nine reactions (*n *= 9) form a chemical event of multi-step reactions. The dynamics of variables × = (*L, X *) is described by the following reactions together with the corresponding propensity functions

(14) $$\begin{array}{ccc} \text{Reaction } & \text{Propensity function} & \\ DNA\overset{k_{1}}{\;\to }\;\left(9L,X\right) & a_{1}=k_{1},\\ \left(X,L\right)\overset{k}{\;\to }\;\left(X,L-1\right) & a_{2}=k\cdot X\cdot \left(1-f\left(L,X,9\right)\right),\\ \left(X,L\right)\overset{k}{\;\to }\;\left(X,L-1,\;L-1\right) & a_{3}=k\cdot X\cdot f\left(L,X,9\right). \end{array}$$

Using the assumption *s*_2 _= . . . = *s*_10_, the rate constant *k *(14) is the harmonic mean of rate constants *s*_2_, . . . , *s*_10_, given by

(15) $$k=\frac{8}{\sum_{i=2}^{10}\frac{1}{s_{i}}}=s_{i}.$$

Next we used the proposed two-variable model to give more accurate simulations. We first estimated the degradation rate constant *k *and optimal initial total length of transcripts. We have also estimated the degradation rate constant *k *by assuming that the total initial length is a half of the maximal total length (*L *= *nX */2), which is termed as the averaged total length. To reduce the computing time, we first estimated parameters in the ODE model (12) using different initial transcript numbers (*X*_0 _= 5, 10, 20, . . . , 100). Table 2 suggests that the variation between the estimate rate constant *k *was very small for different initial mRNA numbers. Similar observation is applied to the ratio of the optimal initial total length to the maximal total initial length, namely *L*_0_/(*nX*), for the tests with different initial mRNA numbers. Thus our results suggested that the estimated model parameters are independent to the initial mRNA copy numbers.

**Table 2:** Estimated parameters for the stochastic model of RPL30 and ACT1 mRNA degradations (Ratio = *L*_0_*/nX*).

*ACT1 *construct				*RPL30 *construct		
*X*_0_	Rate *k*	*L*0	Ratio	Rate *k*	*L*0	Ratio
*m *= 5	0.1150	19	0.4222	0.1710	24	0.5333
*m *= 10	0.1110	37	0.4111	0.1660	47	0.5222
*m *= 20	0.1130	75	0.4167	0.1680	95	0.5278
*m *= 30	0.1130	112	0.4148	0.1670	142	0.5259
*m *= 40	0.1120	149	0.4139	0.1680	190	0.5278
*m *= 50	0.1120	186	0.4133	0.1670	237	0.5267

Using the estimated model parameters of the case *X*_0 _= 100, simulation results for the two constructs in Figure 5 (*A*) and (*B*) show that the two-variable model provides more accurate description of the mRNA degradation dynamics than the one-step model, in particularly for that in the first 25 minutes. For the *ACT1 *construct in Figure 5 (*A*), the optimal length number with ratio 0.412 gave more accurate simulation than the averaged length number. However, in Figure 5 (*B*) for the *RPL30 *transcript, the difference between the simulations using two different length numbers is small. In this case, the optimal ratio is 0.525, which is very close to 0.5.

To further examine the accuracy of the two-variable model, we used the stochastic model to simulate the mRNA dynamics using different initial transcript numbers. For each initial mRNA number, we generated 10, 000 simulations and then calculated the averaged mRNA numbers of all stochastic simulations. For both constructs in Figure 5 (*C*) and (*D*), our results show that there is small difference between the simulations using *X*_0 _= 5 and *X*_0 _= 10. However, there is not any significant difference between simulations when the initial mRNA number is larger than 10.

**Figure 6:**
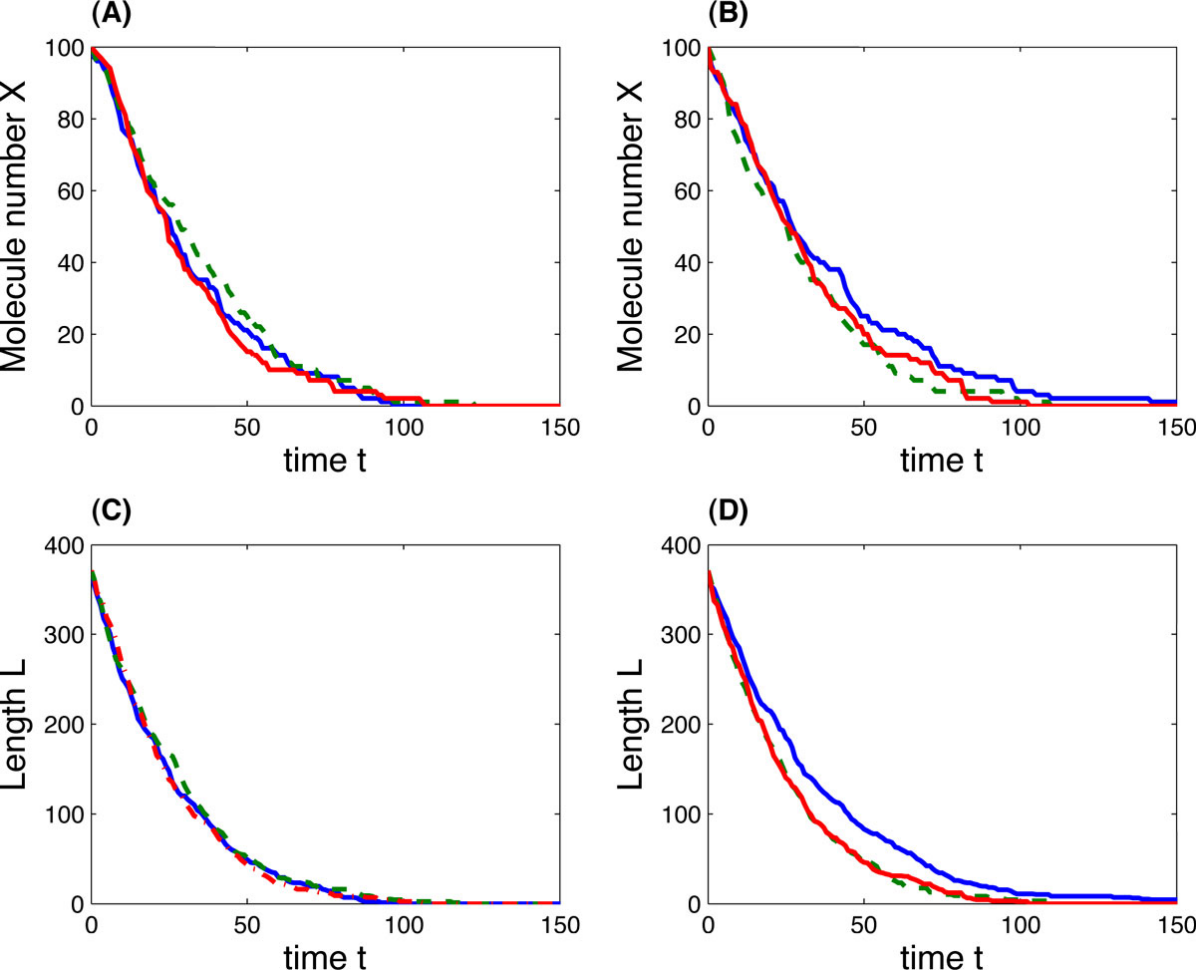
**mRNA degradation dynamics of gene ***RPL30 ***construct ***ACT1 ***in single cells**. (*A, C*) three simulations of *X *and *L *values over *t *for the detailed multi-step reaction model. (*B, D*) three simulations of *X *and *L *values over *t *for the two-variable model. For the detailed multi-step model, rate constants are *s*_1 _= 0, *s*_2 _= . . . = s_10 _= 0.112, and initial molecular numbers are ([*A*], [*B*], [*BC*1], . . . , [*BC*7]) = [4,3,3-5,15,20,20,15,15]. For the two-variable model, rate constant is *k *= 0.112, initial conditions (*X*_0_, *L*_0_) = (100, 371).

Finally we provided a few stochastic simulations for mRNA degradation dynamics for the construct *ACT*1 in single cells. The rate constants of the detailed model were derived from the two-variable model using the relationship (15); and the initial molecular numbers were randomly selected while the length of the initial molecules matches the length in the two-variable model. When the mRNA synthesis rate is *s*_1 _= 0, Figure 6 shows that the molecular numbers and lengths approaches to zero at the time point around 100. In addition, compared with the simulations of the detailed model in Figure 6A and 6C, the two-variable model generates simulations with more fluctuations in Figure 6B and 6D. After the time point 100, more simulations of the two-variable model still have non-zero molecular numbers.

## Conclusions

This work represents an attempt to use simplified mathematical models to describe complex biological systems. Concentrating on the chemical events of multi-step reactions, we proposed a new concept (e.g. the length of a molecule) as an additional measure to characterize system dynamics. The length of a molecule is defined as the location of a molecule in the multi-step reactions. Using the total molecule number and total length of molecules, we proposed a two-variable model to reduce the complexity of the multi-step reactions. The major contribution of this work is to design a nonlinear function that represents the probability of the firing of the last reaction in the multi-step reactions. To calibrate this probability function, we proposed a stochastic simulation method to calculate the probabilities of various system states. Numerical results suggested that this probability is dependent on the number of reaction steps but independent of the total molecule number, which suggested that we were able to design a simplified model based on the network structure. Then our proposed two-variable model was applied to simulate the dynamics of mRNA degradation using experimentally observed data. Numerical results suggested that the length of molecules, which is approximately a half of the maximal length initially, played an important role in realizing experimental data. The potential future work includes the application of the two-variable model to other multi-step reaction systems such as gene expression and telomere length regulation. In addition, the refinement of the two-variable model, such as the accuracy of the probability function, would also be very interesting.

## Methods

### Simulation algorithm for the probability function

To find the probability for the firing of the last reaction in the multi-step reactions (1), we first designed a Monte-Carlo method to numerically calculate the probability function *f*(*X, L, n*) based on the given *X *and *n*. By the law of total probability, the formation of probability that the final reaction occurs given by any L and × is defined as following:

$$f\left(X,L,n\right)=\overset{X}{\underset{j=0}{\sum }}P\left(R_{n}|B_{n}=j\right)\;.\;P\left(B_{n}=j|L,X\right),$$

where *R_n _*represents the occurrence of last reaction $B_{n}\overset{k_{n}}{\underset{}{\to }}P$. Based on the total molecule number *X*, we calculate the probability

$$P\left(R_{n}|B_{n}=j\right)=\frac{j}{X}.$$

The major part of this algorithm is to find frequency of the event *B_n _*= *j *based on the given length *L *and total molecule number *X*, which is explained as the following algorithm I.

*Algorithm I*

1. Set the total number of molecule *X *, number of reactions *n*, and initial full length *L*_0 _= *nX*.

2. Based on the following 10, 000 Monte-Carlo simulations, calculate the frequency *freq*(*B_n _*= *j*|*L, X*) for *X *molecules with total length *L *having *j *molecules, where *j *= 0, 1, . . . *X *and each of the molecule with length 1:

(a) Consider *X *molecules with full length. Denote the length of the *i*-th molecule as *l_i _*with *i *= 0, 1, . . . *X*.

(b) Use a random number *r *~ *U *(0, 1) to select one molecule, with index *j*. If the length of that molecule *l_j _*> 1, reduce its length by 1, namely *l_j _*= *l_j _*− 1; if *l_j _*= 1, then repeat this step until finding a molecule with length greater than 1.

(c) Repeat step (b) for (*L*_0 _− *L*) times to get a set of molecules with total length *L*.

(d) Count the number of molecules in this set with length 1, denote as *i*, then update

$$freq\left(B_{n}=i|L,X\right)=freq\left(B_{n}=i|L,X\right)+1.$$

(e) Repeat steps (*a*) ~ (*d*) for 10,000 times.

3. The probability for the last reaction firing is obtained by

$$f\left(X,L,n\right)=\overset{X}{\underset{j=1}{\sum }}\frac{freq\left(B_{n}=j|L,X\right)}{10000}\times \frac{j}{X}.$$

### Ordinary differential equation model

The most widely used approach to study chemical reaction systems is deterministic model using ordinary differential equations. The approach is valid if the copy numbers of chemical species in the system are large. To confirm the probability function *f*(*X, L, n*) derived from stochastic simulations, we designed a deterministic model of ODEs for the multi-step chemical reaction system (1), given by

(16) $$\begin{array}{ccc} \frac{dB_{1}}{dt} & = & -k_{1}B_{1},\\ \frac{dB_{2}}{dt} & = & k_{1}B_{1}-k_{2}B_{2},\\ & \vdots & \\ \frac{dB_{n}}{dt} & = & k_{n-1}B_{n-1}-k_{n}B_{n},\\ \frac{dP}{dt} & = & k_{n}B_{n}. \end{array}$$

Using the total molecule number *X*(= *B*_1 _+ . . . + *B_n_*) and the total length of the molecules *L*(= *B_n _*+ 2*B*_*n*−1 _+ . . . + *nB*_1_), we have a simplified model of the above ODE system

(17) $$\begin{array}{c} \frac{dX}{dt}=-kB_{n},\\ \frac{dL}{dt}=-kX, \end{array}$$

where *k *is the harmonic mean of the rate constants *k*_1_, . . ., *k_n _*(19), and *kB_n _*represent the probability of molecule degradation which is represented by the probability function *f *(*X, L, n*). Using the notations of stochastic simulation, the ODE model with the length of molecules is given by

(18) $$\begin{array}{c} \frac{dX}{dt}=-kX{\left(1-\frac{L-X}{X\left(n-1\right)}\right)}^{q},\\ \frac{dL}{dt}=-kX. \end{array}$$

For a given initial condition *B_i_*(0), we obtained the analytical solution of the detailed system (16) and then solved the two-variable model (18) numerically using a stiff ODE solver *ode*23*s *in MATLAB. We tested the solution of the two-variable model with different values of *q *based on different system conditions ranging from *n *= 5, 10, 15 as well as *X *= [5 10 50 100 200 500]. For each system condition, we selected the optimal value of q with which the two-variable model (18) generates simulation that is very close to that of the detailed ODE model (16). Finally we find the relationship between the value of *q *and system condition (*X, L, n*) by using a regression method [38].

### An algorithm for simulating systems including two-variable model

The SSA is a general framework for simulating biochemical reaction systems. Now we propose an algorithm to incorporate the two-variable model into the SSA. It is assumed that a chemical reaction system is a well-stirred mixture at constant temperature in a fixed volume Ω. This mixture consists of *N *molecular species {*S*_1_, . . . , *S_N_*} that chemically interact through *M *reaction channels {*R*_1_, . . . , *R_M_*}. The dynamic state of this syetem is denoted as **x **≡ (*x*_1_(*t*), . . . , *x_N _*(*t*))^⊤^, where *x_i_*(*t*) is the molecular number of species *S_i _*at time *t*. For each reaction channel *R_j _*(*j *= 1, . . . , *M*), a propensity function *a_j _*(**x**) is defined by a given state **x**(*t*) = **x **and the value of *a_j _*(**x**)*dt *represents the probability that one reaction will occur somewhere during the infinitesimal time interval [*t, t *+ *dt*) [14][26][41]. In addition, a state change vector *ν_j _*is defined to characterise the change of molecular numbers due to the reaction *R_j_*. The element *ν_ij _*of *ν_j _*represents the change of the copy number of species *S_i_*. The algorithm for simulating chemical reaction systems with two-variable model is given below.

*Algorithm II*

1. Calculate the values of propensity function *a_j _*(**x**) based on the system state **x **at time t. In particular, for the two-variable reaction with the total molecule number *X *(2) and total length *L *(3), the propensity function is *a_j _*= *kX*, where *k *is the harmonic mean of the rate constants (1), given by

(19) $$k=\frac{n}{\frac{1}{k_{1}}+\cdots +\frac{1}{k_{n}}}.$$

Then the sum of propensity function values is

$$a_{0}\left(x\right)=\overset{M}{\underset{j=1}{\sum }}a_{j}\left(x\right).$$

2. Generate a sample *r*_1 _of the uniformly distributed random variable **U**(0, 1), namely *r*_1 _~ *U *(0, 1), and determine the time of next reaction

$$\mu =\frac{1}{a_{0}\left(x\right)}\text{ln}\frac{1}{r_{1}}.$$

3. Generate another sample *r*_2 _of **U**(0, 1) to determine the index *k *of the next reaction occurring in [*t, t *+ *µ*],

$$\overset{k-1}{\underset{j=1}{\sum }}a_{j}\left(x\right)<r_{2}a_{0}\left(x\right)\leq \overset{k}{\underset{j=1}{\sum }}a_{j}\left(x\right)$$

4. If the k-th reaction is not a two-variable model, update the state of the system by

$$x\left(t+\mu \right)=x\left(t\right)+\nu_{k}$$

Otherwise generate a sample *r*_3 _~ **U**(0, 1) to determine which reaction of the followings will occur,

$$\left(X,L\right)=\left\{\begin{array}{cc} \left(X,L-1\right) & \text{if}\;r_{3}>f\left(X,L,n\right),\\ \left(X-1,L-1\right) & \text{if}\;r_{3}<f\left(X,L,n\right), \end{array}\right)$$

where *f *(*X, L, n*) is the probability of the firing of the last reaction. Then the system is updated.

5. Go back to step 1 if *t *+ *µ *≤ *T*, where *T *is the end time point. Otherwise, the system state at *T *is *x*(*T*) = *x*(*t*).

## Competing interests

The authors declare that they have no competing interests.

## Authors' contributions

TT conceived and designed the study. QW and TT developed algorithms and carried out research. QW, KS, TZ and TT analyzed the data, interpreted the results and wrote the paper. All authors edited and approved the final version of the manuscript.

## Declarations

The publication costs for this article were funded by the corresponding author.

This article has been published as part of *BMC Systems Biology *Volume 7 Supplement 4, 2013: Selected articles from the IEEE International Conference on Bioinformatics and Biomedicine 2012: Systems Biology. The full contents of the supplement are available online at http://www.biomedcentral.com/bmcsystbiol/supplements/7/S4.
